# Supplementary material for: Luteolin Induces Nrf2 Activity in C2C12 Cells: Implications for Muscle Health
Source: Int J Mol Sci. 2025 Apr 25;26(9):4092. doi: 10.3390/ijms26094092 (PMC12071863; doi:10.3390/ijms26094092)
Supplement: Supplementary file 1 [file ijms-26-04092-s001.zip › ijms-3579206-supplementary.pdf]

Fig.S1

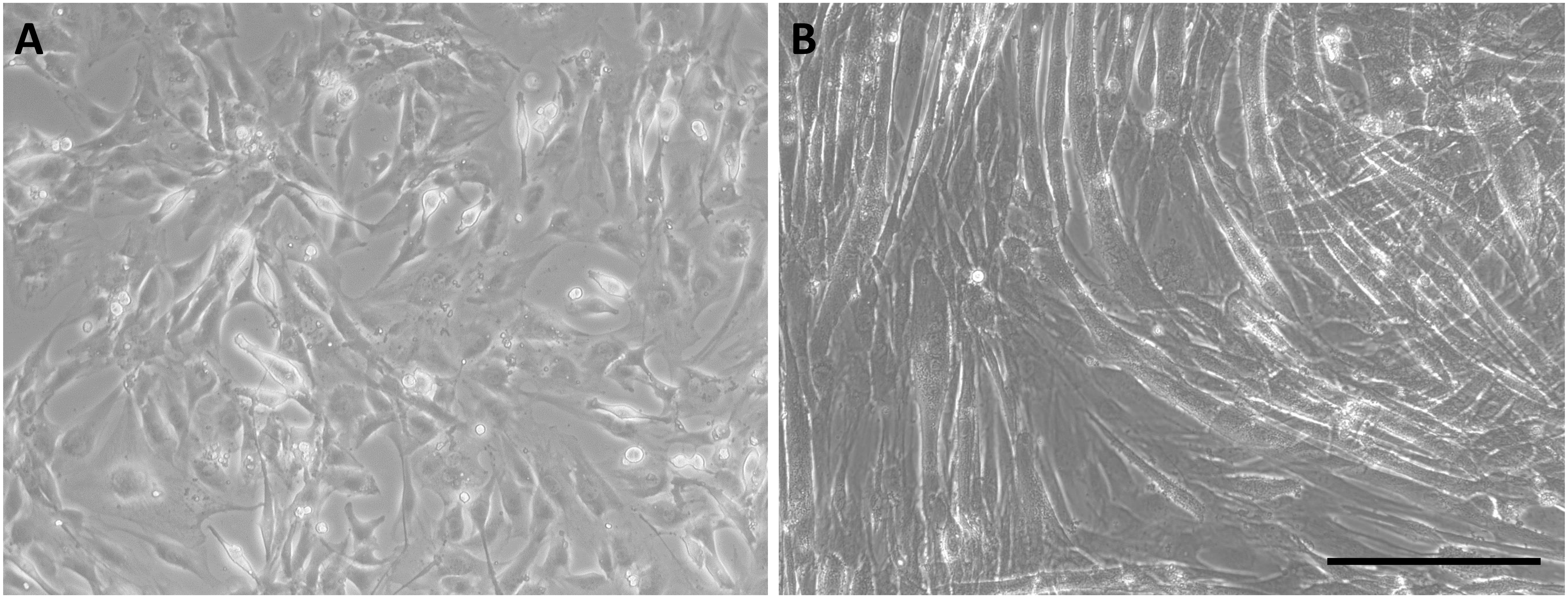

Figure S1 Morphology of C2C12 cells. **:** A) in proliferating state and B) in differentiated state with microtubules. Scale bar represents 100 μm.

Fig.S2

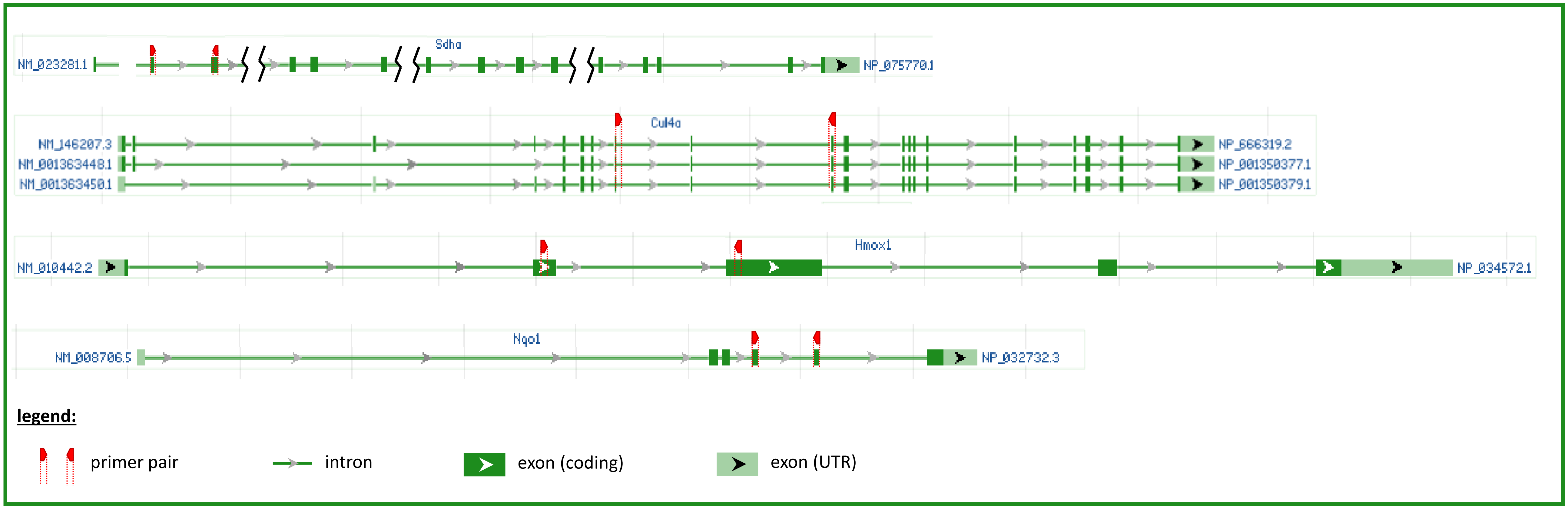

Figure S2 Primer design scheme indicating primer binding sites of the included transcripts. Exons are shown as green boxes, while UTR regions are light green instead of dark green. Introns are presented as lines with arrowheads (longer introns are interrupted to improve clarity of the depiction). Primer pairs are shown as red arrowheads with dotted lines indicating binding on the transcript as well as splice variants.

Table S1

Table S1 Cycle protocol for RT-qPCR

**Stage**	**Temperature [°C]**	**Time [min]**
Hold Stage	50	02:00
	95	10:00
PCR Stage (40x)	95	00:15
	Primer dependent	00:30
	72	00:15
Melt curve stage	95	00:15
	60 (+ 0,1/sec)	01:00
	95	00:15

Table S2

Table S2 Reference genes (REF) and genes of interest (GOI) used in the study.

Target type	Gene	Accession number	Primer sequence	Annealing Temp TA [°C]	Melting Temp TM [°C]	Amplicon length [bp]
REF	Cul4a	NM_001363450.1	5’ TGATGCAGGACAGGGAGGTTC ‘3	59	79.8	123
			3’ CCACACAGGCAATCAACGGT ‘5			
	Sdha	NM_023281.1	5’ GGAACACTCCAAAAACAGACCT ‘3	60	77.9	106
			3’ CCACCACTGGGTATTGAGTAGAA ‘5			
GOI	Hmox1	NM_010442	5’ AAGCCGAGAATGCTGAGTTCA ‘3	61,5	78.5	100
			3’ GCCGTGTAGATATGGTACAAGGA ‘5			
	Nqo1	NM_008706	5’ AGAGAGTGCTCGTAGCAGGAT ‘3	61,5	78.9	103
			3’ CTACCCCCAGTGGTGATAGAAA ‘5			

Fig.S3

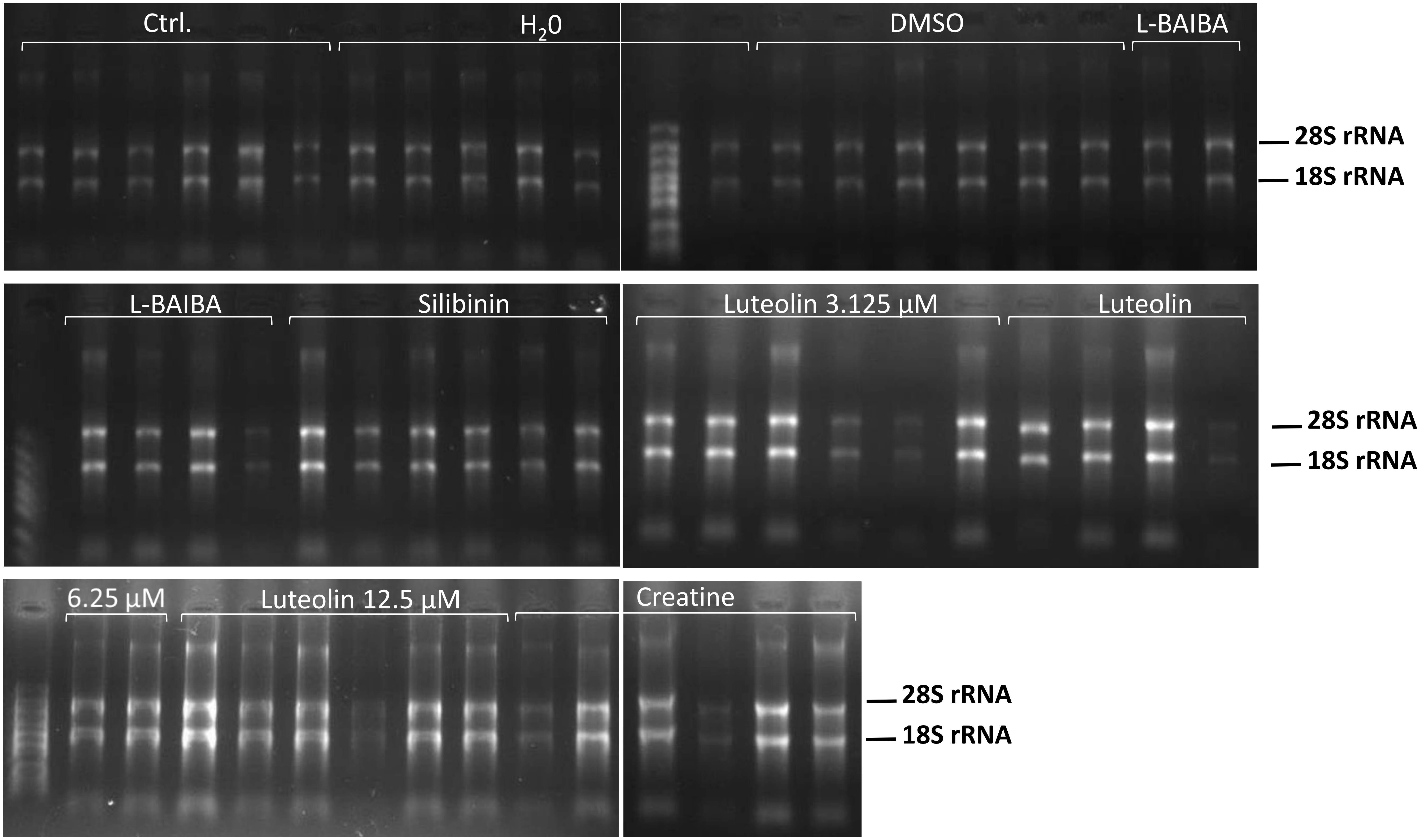

Figure S3: RNA integrity testing of proliferating C2C12 cells by denaturing agarose gel electrophoresis. All RNA samples were prepared with 2x RNA loading dye (Thermo Fisher Scientific; Waltham, Massachusetts, USA) and denatured by heating for 10 min at 70°C. Electrophoresis was performed at a voltage of 6V/cm2 for 40 min in 1x MOPS buffer (0.02 morpholinopropanesulphonic acid, 5 mM sodium acetate, 0.5 mM EDTA, pH 7.0).

Fig.S4

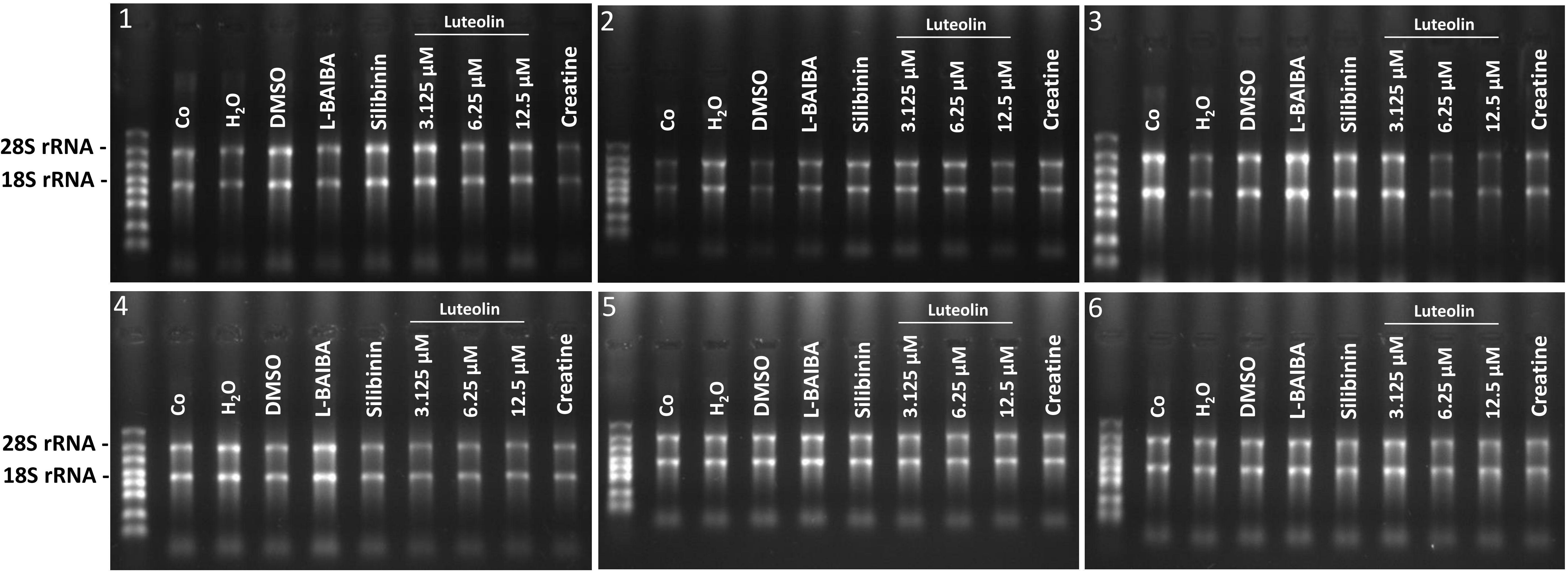

Figure S4: RNA integrity testing of differentiated C2C12 probes by denaturing agarose gel electrophoresis. All RNA samples (Probes 1 – 6) were prepared with 2x RNA loading dye (Thermo Fisher Scientific; Waltham, Massachusetts, USA) and denatured by heating for 10 min at 70°C. Electrophoresis was performed at a voltage of 6V/cm2 for 40 min in 1x MOPS buffer (0.02 morpholinopropanesulphonic acid, 5 mM sodium acetate, 0.5 mM EDTA, pH 7.0).

Fig.S5

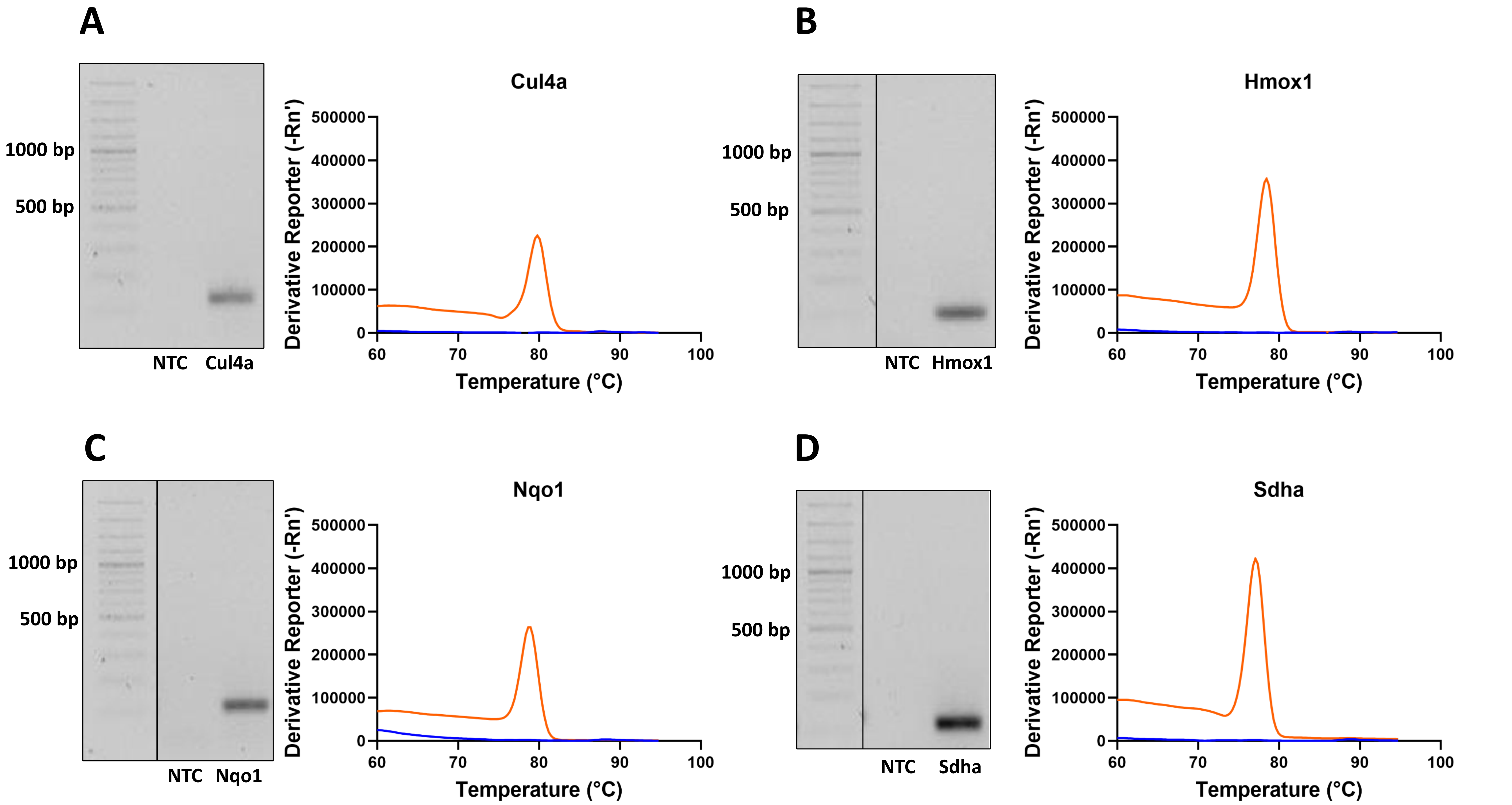

Figure S5 Melt curve analyses and DNA integrity testing by gel electrophoresis separation of qPCR amplicon of proliferating C2C12 cells. Primer specifity was confirmed by 2% agarose gel electrophoresis and melting curve analysis. Reference genes (A and D) and target genes (B and C). Gel shows DNA ladder, nuclease-free water as negative control (NTC) and exemplary qPCR sample with respective gene name below. The black line indicate a separation of the gel. On the right is the corresponding melting curve of the NTC (blue) and exemplary amplicon (orange) shown. Amplicon length and melting temperature for each primer set are summarized in Table S1.

Fig.S6

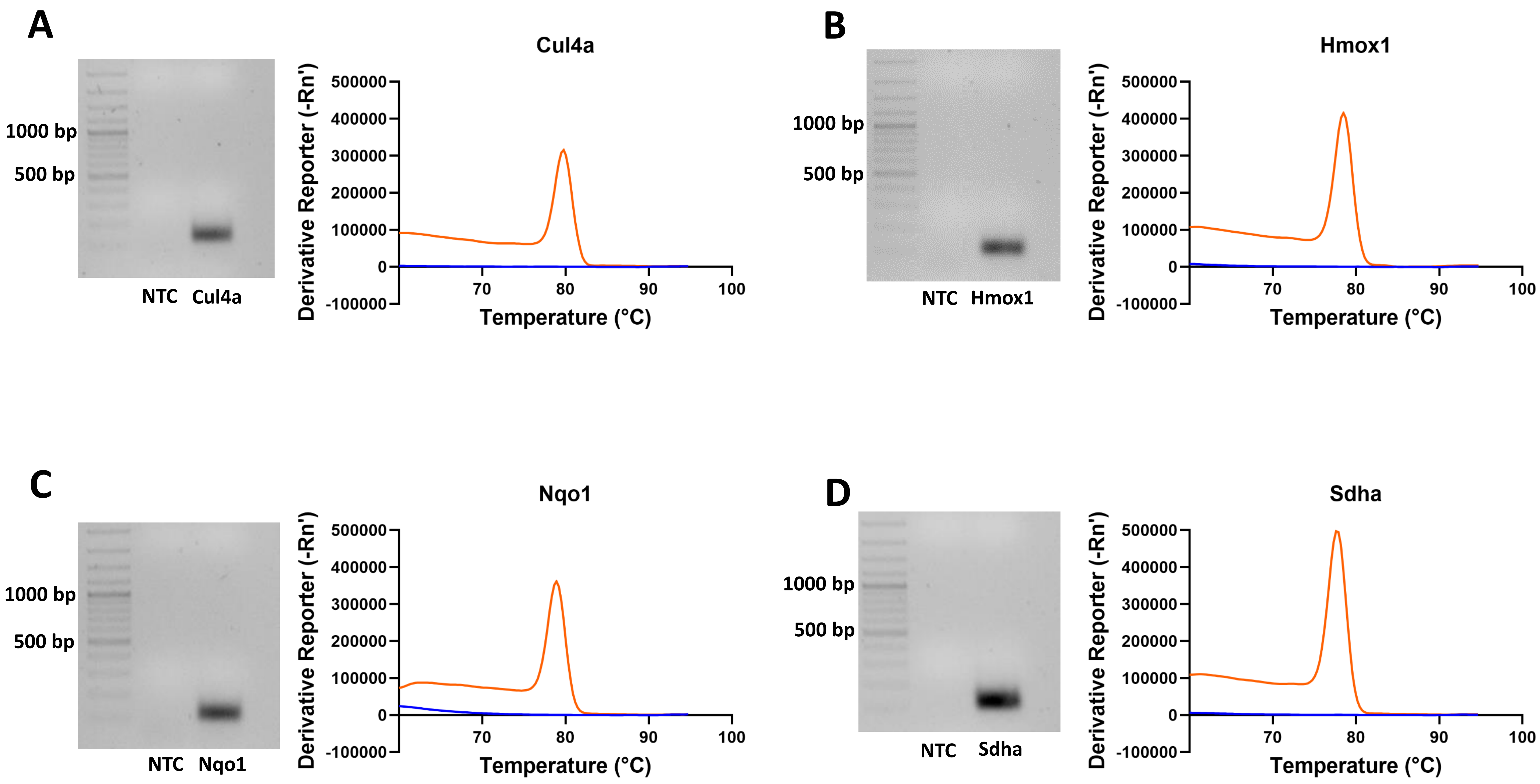

Figure S6 Melt curve analyses and DNA integrity testing by gel electrophoresis separation of qPCR amplicon of differentiated C2C12 cells. Primer specifity was confirmed by 2% agarose gel electrophoresis and melting curve analysis. Reference genes (A and D) and target genes (B and C). Gel shows DNA ladder, nuclease-free water as negative control (NTC) and exemplary qPCR sample with respective gene name below. The black line indicate a separation of the gel. On the right is the corresponding melting curve of the NTC (blue) and exemplary amplicon (orange) shown. Amplicon length and melting temperature for each primer set are summarized in Table S1.
